# The role of mediation in solving medical disputes in China

**DOI:** 10.1186/s12913-020-5044-7

**Published:** 2020-03-18

**Authors:** Mengxiao Wang, Gordon G. Liu, Hanqing Zhao, Thomas Butt, Maorui Yang, Yujie Cui

**Affiliations:** 1 National School of Development, Peking University, Beijing, 100871 China; 2 People’s Mediation Committee in Guangdong, Guangzhou, 510095 China; 3 Sichuan Development Center for Healthy Aging, Chengdu, 610094 China; 4 Department of Economics, Louisiana State University, Baton Rouge, 70820 USA; 5 China Hospital Development Institute, Shanghai Jiao Tong University, Shanghai, 200025 China

**Keywords:** Medical dispute, Compensation, Duration, Medical mediation, China

## Abstract

**Background:**

Medical litigation represents a growing cost to healthcare systems. Mediation, arbitration, and other alternative dispute resolution (ADR) methods are increasingly used to help solve the disputes and improve healthcare satisfaction. In China, the increasing number of medical disputes has contributed to concern for the safety of physicians and mistrust between physician and patients resulting in ADR processes being established in several provinces in recent years. Our aim was to describe and explain the impact of this new mediation process in the Chinese healthcare system.

**Methods:**

Our study investigated mediation practices in China using case-level data from 5614 mediation records in Guangdong Province between 2013 and 2015. We investigated how the resolution success as well as the compensations are associated with the case characteristics using regression analysis.

**Results:**

Among the cases analyzed, 1995 (41%) were solved with agreement through mediation, 1030 were closed by reconciliation, 559 were closed by referring to court and 1017 cases were withdrawn after mediation. Five hundred five *Yinao* cases were solved with the help of mediators on the spot. We find that mediation solved about 90% of medical disputes under present mechanisms, while more police support is needed to cope with *Yinao*. The average compensation of mediation is CNY60,200 and average length of mediation is 87 days. Longer time taken to reach resolution and more money claimed by patients are associated with lower resolution success rate (*p* < 0.01) and higher compensation levels (p < 0.01).

**Conclusion:**

Our results show the performance of mediation mechanisms in China to help solve medical disputes. ADR plays a role in reducing the need for initiating litigation and may ultimately increase satisfaction with the healthcare system.

## Background

Accompanied by the rapid growth of household income, demand for better healthcare services has been increasing in China, leading to rising expenditure on healthcare. At the same time, a number of organizational reforms on the supply-side have been implemented to improve the quality and efficiency of Chinese healthcare delivery [1, 2]. Despite these improvements, the cost and volume of medical disputes has grown in recent years. Mistrust between patients and physicians has grown [3] and the physician-patient relationship has worsened. Extreme cases of medical disputes can cause great physical, mental and reputational damages to physicians and hospitals, and bring losses and adverse social influences due to negative media coverage. Workplace violence has been shown to contribute to higher levels of occupational stress and lower levels of job satisfaction [4, 5] and even brought psychological problems to physicians [6].

Hospital disputes occur in nearly all departments and most often in tertiary hospitals [7]. One particular case of hospital violence, commonly known as *Yinao* in Chinese, describes organized unemployed gangs who are paid by patient families to create medical disturbances to get compensation for actual or perceived malpractice from hospitals [8]. Through this illegal approach, patients in dispute with hospitals usually expect to get higher compensation compared to other approaches [9]. With the hospital being surrounded by *Yinao* gangs, hospital operations such as diagnosis and treatment are disrupted and hospital staff, equipment and facilities are at risk.

In one recent example, on July 12 of 2018, a female doctor was stabbed by three gangsters in Tianjin and died later in hospital. None of the three gangsters were patients of the doctor [10].

To tackle this problem, China has made a number of changes to laws and regulations. Several Provisions of the Supreme People’s Court on Evidence in Civil Procedures meant that medical damage lawsuits started to be judged based on reversed onus from 2002. However, this created more tension between physicians and patients and made physicians more defensive and spend more time on medical record keeping [11] with implications for the quality of medical services while contributing to increase in health care costs [12]. In 2010, the Tort Liability Law was implemented and the reversed onus was to some degree reduced for healthcare providers [13].

To deal with more serious disputes, extreme forms of *Yinao* like “setting up a mourning hall”, “burning paper money” (a Chinese traditional way to memorize the dead at a funeral) to disrupt hospital operations and so forth can constitute crimes [14]. This was formalized by Amendment IX to the Criminal Law of China in 2015 whereby crowds who are assembled to disrupt hospital services will be sentenced with serious consequences.

Most recently, the government has begun to advocate resolution of disputes by alternative mechanisms. On July 31, 2018, Prime Minister Li Keqiang signed “Medical Disputes Prevention and Treatment Regulations”, which further emphasized the prevention and resolution of medical disputes and protection of both physicians and patients.

Medical disputes can be resolved through litigation [15, 16] or, where alternative dispute resolution (ADR) methods exist, through mediation or arbitration [17, 18]. Facing the scarcity of both medical and legal resources, China has advocated mediation as an ADR approach to deal with medical disputes. Gradually, mediation has been adopted in many provinces and cities, such as Guangdong Province, Tianjin City and Hainan Province. Studies on approaches to deal with medical disputes are more focused on litigation [19, 20], a few have analyzed a small sample of mediation cases without highlighting the role of mediation in coping with *Yinao* [21, 22].

The contribution of this study is threefold. First, this study provides an analysis of mediation practices in Guangdong, the largest province in China, with 5614 case-level data and a detailed description of the mediation mechanism. Second, we analyze the medical disputes handled through mediation-based cases from 2013 to 2015 in China and investigate the factors that influence successful mediation and levels of compensation. Third, our analysis deals with not only the common medical disputes but also *Yinao* cases. With the information of 505 *Yinao* cases, this study investigates the unique features of *Yinao*.

Our study aims to quantitatively describe and explain the outcomes of mediation in China to provide policy recommendations for hospital risk management and safety enhancement of both patients and physicians.

## Methods

### Mediation in China

According to the People’s Mediation Law, the People’s Mediation Committee is under the supervision of the Judicial Administration department, and mediates disputes for free. The agreement after mediation has legal effect but no power of law enforcement. In order to be legally binding, patients or hospitals must seek judicial confirmation. Without this confirmation, if either side defaults on the agreement, mediators will advise them to choose other ways to deal with the dispute.

Guangdong province in China had a GDP of 8.09 trillion CNY and a population of 110 million people in 2016, making it the largest province measured by wealth and population [23]. Most citizens live in urban areas (69.2%, 2016). In 2017, the migrant population in Guangdong reached 41 million, which adds difficulty to public governance and conflict resolution.

**Fig. 1:**
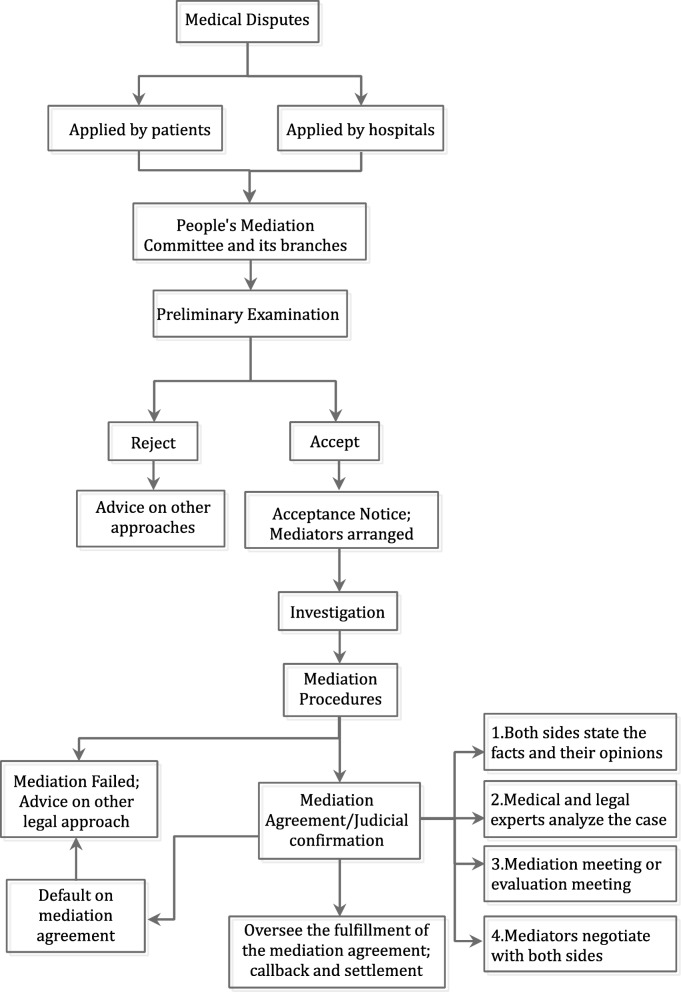
The mediation process of People’s Mediation Committee in Guangdong

Guangdong People’s Mediation Committee (PMC) was established in 2010, and its service region has expanded to 16 out of 21 cities by 2015. Guangdong’s PMC operates according to the principles of “Fairness, Impartiality, Neutrality, Timeliness, and Convenience”. It built a mechanism combining mediation, compensation and dispute prevention, which actively responses to *Yinao* and aims to settle medical disputes outside hospitals to maintain normal diagnosis and treatment order. To mediate disputes between health care facilities and patients, the mediators usually have educational backgrounds such as medicine, law, and psychology and they receive annual training to improve their mediation skills. The mediation process is also supported by both medical and legal experts and most of the medical experts have a job title of deputy director or above. Guangdong PMC has 46 branches in Guangdong Province and its expert group (including doctors, nurses and lawyers) has more than 2000 members. Figure 1 depicts the mediation procedure in Guangdong. Usually the patient or medical institution applies for mediation first. Once accepted, the mediator will start by collecting evidence (e.g. medical records). The main procedure of mediation contains four steps: 1), both sides make statements; 2), medical and legal experts analyze the case; 3), experts are invited for meetings regarding to the specific case (e.g. clinic department, treatment or nursing); and 4), the mediator negotiates with both patient and hospital sides based on the opinions of the experts.

Generally, medical disputes will be handled at the PMC and follow the procedures described above. While for *Yinao* cases, once reported, the mediators will go to hospitals to handle them on site.

### Data sample

**Fig. 2:**
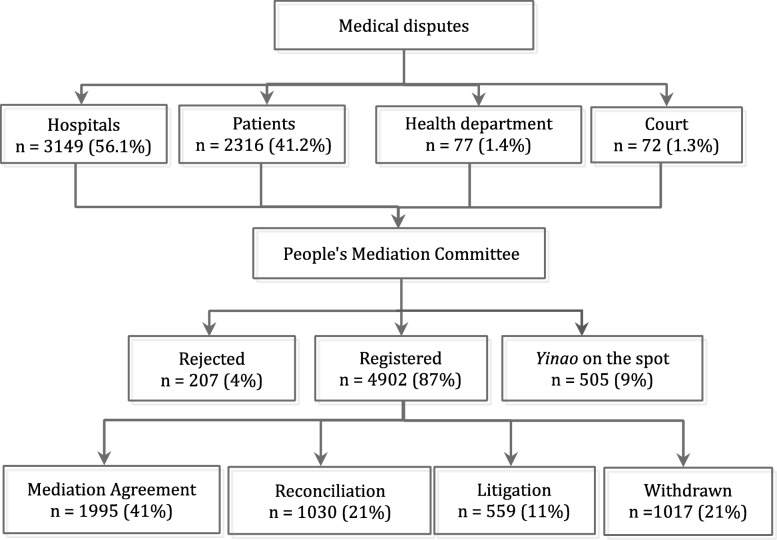
Overview of the approaches and outcomes of mediation. Notes: We analyzed 5614 mediation cases in total, including 5109 cases settled at the People’s Mediation committee and 505 *Yinao* cases settled on the spot

We used data from Guangdong PMC, which contains information on case-level medical disputes, including cause, duration, and hospital characteristics. Our sample included 5614 cases settled during 2013–2015 in Guangdong Province. Ninety-seven percent of mediation cases were reported by hospitals and patients (Fig. 2). The cases referred by health departments or courts account for only 1 % each. Overall, 4902 out of the 5614 cases (87%) were registered and 207 cases (4%) were rejected after preliminary examination. 505 *Yinao* cases (9%) were handled on the spot.

The outcomes of the 4902 registered mediation cases closed during 2013–2015 consisted of four types: 1995 cases reached mediation agreement (41%); 1030 cases resulted in reconciliations between hospitals and patients (21%); 559 cases proceeded to litigation (11%); 1017 withdrawal of cases (21%). The outcome of 301 (6%) cases were missing.

### Regression method

We used logistic regression to analyze the mediation success and generalized linear regression to analyze compensation of mediation. Given the positively skewed distribution of compensation, we use a generalized linear regression model with gamma distribution [24]. We have investigated the relationship of mediation success and compensation amount with duration of the case, first claim by patients, age of patients, controlling causes of medical disputes, hospital classification (primary, secondary, and tertiary), consequences and year effects.

## Results

### Characteristics of mediation cases

The number of medical disputes increased from 1234 in 2013 to 2293 in 2014 and remained above 2000 in 2015 (Table 1). The mean age of patients in our sample was 37.6 and the mean claim amount was CNY357,900. The top three causes of disputes were: different opinions on responsibility (for example, patients would ask that hospitals take major or all responsibility for the unsatisfactory medical outcome) (54%), the skill of physicians or nurses (27%) and discontent with nursing and medicine (7%). While medical accidents (the unexpected and unintentional medical harm, usually out of limited medical conditions and skills) (5%) and hospital management issue (4%), and lack of informed consent (3%) accounted for small proportions of the cases.

**Table 1:** Characteristics of 5614 Mediation records, China, 2013–2015

Category		Category	
**Closure date-no.(%)**	5614	≤10	297(10)
2013	1234(22)	> 10,≤100	1110(39)
2014	2293(41)	> 100,≤500	847(30)
2015	2087(37)	> 500	584(21)
**Main cause-no.(%)**	4927	**Hospital grade-no.(%)**	5614
Responsibility	2656(54)	Primary	943(17)
Skills	1336(27)	Secondary	2627(47)
Nursing and Medicine	333(7)	Tertiary	2044(36)
Medical Accidents	249(5)	**Medical Specialties-no.(%)**	5072
Management problem	190(4)	General surgery	1553(31)
Informed consent	163(3)	Obstetrics and gynecology	1290(25)
**Time from dispute to closure (days)-no.(%)**	4953	Internal medicine	824(16)
Mean	87	Pediatrics	355(7)
≤1 month	1798(36)	Ophthalmology and otorhinolaryngology	146(3)
> 1 month,≤3 month	1425(29)	Oncology	73(1)
> 3 month,≤6 month	1054(21)	Others	831(16)
> 6 month,≤1 year	536(11)	**Type of medical facility-no.(%)**	5614
> 1 year	140(3)	General hospital	3792(68)
**Closed with compensation-no.(%)**	2941	Township hospital	546(10)
Mean (CNY1,000)	60.2	Traditional Chinese Medicine hospital	522(9)
≤10	1203(41)	Maternal and Child Health hospital	493(9)
> 10,≤100	1328(45)	Specialty hospital	261(5)
> 100,≤500	369(13)	**Severity-no.(%)**	5590
> 500	41(1)	Death	2305(41)
**First claim by patients-no.(%)**	2840	Disability	429(8)
Mean (CNY1,000)	357.9	Others	2856(51)

**SOURCE** Authors’ analysis of mediation data for 2013–2015

More than half of the cases closed with compensation to patients. The average compensation was CNY60,200, which is much less than the amount claimed by patients. Most cases (86%) closed with compensation of less than CNY100,000. Only 1 % of cases settled with compensation above CNY500,000.

The average length of time between registration and closure was 87 days. 1798 (36%) of the medical disputes were settled within 1 month, 65% of them were solved within 3 months and 86% were closed within half a year. 140 (3%) of the cases lasted for more than 1 year.

Our sample consists of 2044 tertiary hospitals (36%), 2627 secondary hospitals (47%) and 943 (17%) primary hospitals. In China, the hospital accreditation system posed lower bounds on the number of beds for each of the hospital grades, of 20, 100, and 500 beds for primary, secondary and tertiary hospitals respectively [25].

General hospitals were more frequently involved in medical disputes (3792 cases, 68%), followed by township hospitals (546 cases, 10%). Traditional Chinese medicine hospitals and maternal and child health hospitals accounted for 9% of all cases each. Departments like general surgery, obstetrics and gynecology, internal medicine and pediatrics are more often involved with medical disputes, accounting for almost 80% of the cases in total.

About half of the cases were related to injury to patients. We find that death is the cause of a large proportion (41%) of these cases compared to disability (8%).

Table 2 illustrates the features of *Yinao* cases that are reported to People’s Mediation Committee and handled by mediators on the spot. During 2013–2015, there were 505 cases in total, which is much more than the number of cases reported by the media. The sudden increase of *Yinao* cases from 14 in 2013 to more than 200 in later years is mainly because People’s Mediation Committee started to record *Yinao* cases separately and more clearly in 2014. Overall, tertiary and secondary hospitals are more common locations for *Yinao*. On average, 26 people take part in *Yinao* and some of them are reported as “professional *Yinao*”, which means they were hired by the patient side and making a living by taking disruptive actions in hospitals to make hospitals to pay to the patients. The chaos lasts for 5 hours on average and can be more than 10 hours in some extreme cases. Most *Yinao* cases are related to death of patients, which accounts for 79% of all the *Yinao* cases.

**Table 2:** *Yinao* cases mediated on the spot, China, 2013–2015

Category		Category	
**Yinao date-no.(%)**	505	> 20,≤50	168(35)
2013	14(3)	> 50	37(8)
2014	237(47)	**Yinao Duration (hours)-no.(%)**	456
2015	254(50)	Mean	5
**Hospital level-no.(%)**	505	≤5	274(60)
Tertiary	165(33)	> 5,≤10	135(30)
Secondary	232(46)	> 10	47(10)
Primary	108(21)	**Severity-no.(%)**	505
**No. of participants-no.(%)**	482	Death	399(79)
Mean	26	Disability	17(3)
≤10	136(28)	Others	89(18)
> 10,≤20	141(29)		

**SOURCE** Authors’ analysis of mediation data for 2013–2015

### Regression analysis on success rate of mediation

We use logistic regression model to analyze the outcomes of mediation. Since ADR is often viewed as complementary to lawsuits, we classified mediation agreement, reconciliation and withdrawn cases as successful, indicated by one in our model. Litigation outcome is represented by zero. Table 3 shows the results of our logistic regression.

**Table 3:** Logistic regression analysis of the success rate of mediation, China, 2013–2015

	(1)	(2)	(3)	(4)
VARIABLES				
Duration of case	0.998***	0.998***	0.997***	0.997***
	(0.000)	(0.000)	(0.000)	(0.001)
First claim by patients				0.993***
				(0.002)
Age of patients	1.001	1.002	1.001	1.000
	(0.002)	(0.002)	(0.002)	(0.003)
Main Cause				
Medical Accidents	1.357	1.515	1.730**	2.738**
	(0.339)	(0.387)	(0.451)	(1.154)
Skills	0.948	0.885	1.009	1.119
	(0.103)	(0.099)	(0.116)	(0.175)
Nursing and Medicine	1.687**	1.605**	1.693**	1.225
	(0.400)	(0.382)	(0.406)	(0.332)
Informed consent	0.713	0.664*	0.655*	0.604*
	(0.170)	(0.162)	(0.162)	(0.182)
Management problem	1.376	1.418	1.467	2.210*
	(0.417)	(0.437)	(0.459)	(0.956)
Hospital level				
Primary	1.836***	1.835***	1.844***	1.484**
	(0.271)	(0.277)	(0.279)	(0.292)
Secondary	2.090***	2.029***	2.047***	1.919***
	(0.217)	(0.214)	(0.218)	(0.277)
Consequence				
Disabled		0.891	0.869	0.926
		(0.143)	(0.141)	(0.203)
Others		2.530***	2.524***	1.926***
		(0.270)	(0.272)	(0.297)
Year				
Year 2014			1.700***	1.848***
			(0.202)	(0.296)
Year 2015			2.649***	2.672***
			(0.348)	(0.480)
Constant	5.604***	3.740***	2.247***	3.371***
	(0.667)	(0.478)	(0.339)	(0.825)
Observations	4313	4313	4313	2525

Robust se in parenthese

*** *p* < 0.01, ** *p* < 0.05, * *p* < 0.1

*Note*: Mediation agreement, reconciliation and withdrawn cases are classified as successful. Litigation cases are classified as unsuccessful

Duration has an odds ratio of 0.998 and is significant, which means for one-day increase in duration the odds of the case solved through mediation decreases by 0.2% given other variables are fixed. Compared to responsibility, the odds ratio of nursing and medicine is positive and significant, which means its chance to be solved through mediation is 68.7% higher than responsibility. With tertiary hospitals as reference, we find that the cases in primary and secondary hospitals are more likely to be solved through mediation and secondary hospital cases have the highest chance to be mediated.

After including the consequence of the cases, column 2 shows that other outcomes are more likely to be solved through mediation compared to cases with death as results. Since other cases are less severe than death or disability cases, it is easier to clarify the cases and for the patients and hospitals to reach agreements.

Considering the trend of mediation, we find that the chance of successful mediation grows year by year. In 2014, the odds is 1.7 and significant, which means the chance of success is 70% higher than 2013. In 2015, the odds is 2.649 and significant, which mean the chance of success is 165% higher than 2013. We can see the success rates of mediation is increasing which may be due to increased experience.

Finally, the first claim of patients is also included in our model. Since patients may not be clear about the compensation amount when they chose mediation, the sample size dropped to 2525 because of missing values of first claim amount. Column 4 shows that for CNY10,000 increase in first claim amount by patients, the odds decrease by 7% given other factors remain fixed. Compared to responsibility, the odds ratio of nursing and medicine becomes insignificant, while the odds ratio of management problem becomes significant at 10% level, which means its chance to be solved through mediation is 121% higher than responsibility. The successful mediation trend grows at a higher magnitude compared to year 2013, reaching 84.8% in 2014 and 167% in 2015.

### Regression analysis on compensation of mediation

Table 4 shows generalized linear model analysis of compensation. Across all the columns, we can see that the coefficient of duration is positive and significant. The coefficient of duration shows that for one-day increase in duration, the compensation can increase by 0.2 to 0.3% (exp (0.002)-1, same below). The coefficient of patients’ age is insignificant all the time.

**Table 4:** Generalized linear regression analysis of the compensation of mediation, China, 2013–2015

	(1)	(2)	(3)	(4)
VARIABLES				
Duration of case	0.003***	0.003***	0.003***	0.002***
	(0.000)	(0.000)	(0.000)	(0.000)
First claim by patients				0.014***
				(0.001)
Age of patients	−0.001	−0.002	−0.002	0.001
	(0.001)	(0.001)	(0.001)	(0.001)
Main Cause				
Medical Accidents	0.124	−0.081	−0.107	− 0.116
	(0.147)	(0.121)	(0.120)	(0.119)
Skills	−0.167**	− 0.016	− 0.042	0.035
	(0.077)	(0.078)	(0.078)	(0.077)
Nursing and Medicine	−0.785***	−0.749***	− 0.771***	−0.530***
	(0.138)	(0.103)	(0.102)	(0.102)
Informed consent	−0.776***	−0.716***	− 0.735***	−0.391**
	(0.227)	(0.199)	(0.201)	(0.174)
Management problem	−0.758***	−0.741***	− 0.758***	−0.464***
	(0.220)	(0.160)	(0.154)	(0.117)
Hospital level				
Primary	−0.167	−0.222**	−0.223**	− 0.241***
	(0.108)	(0.096)	(0.097)	(0.092)
Secondary	−0.213***	−0.216***	− 0.217***	−0.191***
	(0.075)	(0.075)	(0.074)	(0.070)
Consequence				
Disabled		0.016	−0.004	0.097
		(0.110)	(0.109)	(0.098)
Others		−1.256***	−1.261***	−0.841***
		(0.067)	(0.066)	(0.072)
Year				
Year 2014			0.035	0.055
			(0.090)	(0.074)
Year 2015			−0.147*	−0.069
			(0.087)	(0.080)
Constant	1.821***	2.303***	2.339***	1.433***
	(0.088)	(0.090)	(0.106)	(0.121)
Observations	2764	2764	2764	1726

Robust se in parenthese

*** *p* < 0.01, ** *p* < 0.05, * *p* < 0.1

Compared to responsibility, most other causes of medical disputes are compensated less. In column 1, we can see that the compensation of nursing and medicine is 54.4% less than responsibility, followed by inform consent and management problem. Medical accident is associated with higher compensation compared to responsibility, while the result is not significant.

The coefficient of secondary hospital is negative and significant in all models. In column 1, we find that secondary hospitals are compensated 19.2% less than tertiary hospitals on average. After controlling for consequence and year effects, the compensation is about 19.5% less than tertiary hospitals.

After including first claim amount by patients, we find for CNY10,000 increase in the claimed amount, the compensation will increase by 14.1%. In column 4, we find that the coefficient of disabled is insignificant, which means the compensation of disabled is similar to death cases on average. The coefficient for others category is negative and significant, which means the less severe cases will get 56.9% less than death cases on average.

Although the coefficient of year is significant at 10% level in 2015 in column 3, the year effects becomes insignificant in column 4. It is reasonable because the compensation standard doesn’t change that much in different years.

## Discussion

### Mediation as a buffer

Our data shows that 89% of the cases were successfully handled through mediation without going to litigation and the average length of mediation is 87 days, which are comparable to the ADR practice in America and Canada [26]. Our result highlights the importance of communication between different sides. We find that mediation can help avoid “nuisance lawsuits” by offering suggestions to patients and hospitals and encourage them to give up unreasonable demands. 4% of cases were rejected and 11% of cases were withdrawn, which reflects that many cases can be settled after clarification of the medical and legal issues, saving time and resources. Mediation can offer physicians and patients the chance to talk, negotiate and apologize, which may also improve the doctor-patient relationship.

More medical and legal knowledge is helpful for patients to shape a reasonable expectation on the outcome of medical services and compensation amount, which can ease the conflicts between both sides and help build consensus. Our regression analysis results show that both longer duration and higher claim amount are associated with less probability to be solved by mediation and higher compensation. Compared to death cases, less severe cases are more likely to be settled through mediation, and closed with less compensation. It reflects that patients actually have the right idea about the severity of medical services’ consequence, while in general they may initially claim more than the actual compensation amount received.

PMC, as a third-party, has gradually gained popularity. More specifically, the more flexible process and easier access for patients can help save a lot of time and money in contrast with lawsuits [16, 19]. The neutral stance offers PMC a more objective view compared to mediation conducted by health administrative departments [27].

Given all these advantages and to avoid the previous privately and costly settlement between medical institution and patients [28], several regions have implemented special regulations on the ways to solve medical disputes and mediation is almost the first choice for both patients and hospitals there. In 2013, the government of Guangdong Province issued a provincial notice on prevention and resolution of medical disputes, which stated that negotiation between hospital and patients is only appropriate for claims below CNY10,000 [29]. Similarly, Baoji city in Shaanxi Province also set a cap on the compensation for negotiation approach in 2015 [30].

### Hospital management can play a role in dispute prevention and resolution

Studies have shown the possibility to reduce disputes by changing the procedure of dealing with medical disputes [31] and early detection/intervention of medical disputes [32].

In our analysis, we find that tertiary hospitals and departments like surgery, obstetrics and gynecology, and internal medicine are more likely to face medical disputes. According to the requirements of the National Health Commission (previously known as the Ministry of Health) [33], tertiary hospitals are designed to treat patients with serious conditions as well as common diseases and supposed to provide higher-quality of services, so they are more likely to be involved in complex medical disputes with associated higher compensation. Our results show that cases in primary and secondary hospitals are more likely to be solved through mediation and end up with less compensation compared to tertiary hospitals. Surgery, obstetrics and gynecology, and internal medicine departments are the top three departments that are involved in medical disputes. The analysis on main causes show that most of the medical disputes can be attributed to responsibility and skills, it is more demanding for tertiary hospitals and the top three departments to enhance their risk management practices and help improve the skills of relative services.

### More help is needed to handle *Yinao* cases

The average duration (5 h) and number of people participating (26) in *Yinao* depict the chaotic scene faced by hospitals during 2013–2015. In China, legal progress has been made to prohibit *Yinao*. However, we find that most *Yinao* cases are related with the death or disability of patients (82%). Consequently, it may be hard to peacefully resolve *Yinao* and more support should be given to guarantee the safety of physicians. In 2016, we conducted interviews with the mediators and the director of PMC. Based on their working experiences, we found that *Yinao* is not well prohibited and that the police force didn’t go to the spot to calm down the issue every time.

Although mediators can go and help deal with *Yinao* cases, it’s necessary for police department and procuratorate to participate and solve *Yinao* at the time. As the “Medical Disputes Prevention and Treatment Regulations” in 2018 has stated that hospitals should report *Yinao* to the police immediately, more analysis should be done to evaluate whether police forces have the necessary resources to handle *Yinao* cases. The influences of *Yinao* are not just limited within one case. In China, extreme *Yinao* cases will be on the news and generate social media discussion. It is necessary for the media to advocate and emphasize the legal ways to solve disputes instead of just reporting the fact of *Yinao* or violence against physicians.

### Limitations

Our study has several limitations. First, the finding that mediation is time-saving compared to litigation maybe impacted by the observation period. We only included cases that were settled during the observation period so there could be some very long ongoing cases that were not resolved during this time and so the average length of resolution may be under-estimated. Furthermore, some serious cases may bypass the mediation method and choose the legal approach directly. Second, due to data availability, our analysis focuses on the mechanism and results of cases handled through PMC in Guangdong Province, further analysis could include data from other provinces to confirm if our findings in Guangdong apply at the national level. Third, our data didn’t cover the period after 2015, therefore we cannot capture the effects of litigation change before and after *Yinao* was included in Criminal Law in 2015. Since this law change is expected to deter patients from taking illegal actions, future analysis would be valuable to understand its effect on *Yinao* cases in more detail.

## Conclusion

Through the analysis of the mediation committee work in China, we find that mediation can help substantially reduce conflicts between physicians and patients to avoid litigations, thus saving time and money for both parties. While for illegal actions taken by patients like *Yinao*, not only mediators but also the police are needed to fully handle such cases. By setting up a neutral third-party institution like the mediation committee together with an appropriate legal framework, healthcare systems can also better handle the disputes between hospitals and patients to improve the patient-provider relationships and public satisfaction.

## Data Availability

The data that support the findings of this study are available from People’s Mediation Committee in Guangdong but restrictions apply to the availability of these data, which were used under license for the current study, and so are not publicly available. Data are however available upon research request and with permission of People’s Mediation Committee in Guangdong.

## Abbreviations

ADR: Alternative Dispute Resolution
CNY: China Yuan
GDP: Gross Domestic Product
PMC: People’s Mediation Committee
